# CRYing for balance: a repressor lingers through peak circadian transcription

**DOI:** 10.1038/s44318-025-00467-4

**Published:** 2025-05-30

**Authors:** Michael Brunner

**Affiliations:** Heidelberg University Biochemistry Center, Im Neuenheimer Feld 328, 69120 Heidelberg, Germany

**Keywords:** Chromatin, Transcription & Genomics, Neuroscience, Post-translational Modifications & Proteolysis

## Abstract

A new study sheds light on circadian clock protein dynamics.

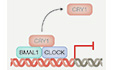

## Teaser

Circadian rhythms in mammals rely on cell-autonomous molecular clocks that operate in many tissues and organs. New research in *The EMBO Journal* quantifies the expression dynamics, distribution, mobility, and turnover of circadian clock proteins in the suprachiasmatic nucleus. The quantitative data obtained from a physiologically relevant tissue will inspire tailored in vitro approaches and help to put these findings into a physiological context to further develop our current model of the circadian clock.

At the organismal level, synchronization of circadian molecular clocks with the environmental day-night cycle is mediated by the suprachiasmatic nucleus (SCN) of the hypothalamus. At the core of the cell-autonomous circadian clock is the heterodimeric transcriptional activator BMAL1/CLOCK, which orchestrates the rhythmic expression of numerous clock-controlled genes including its direct negative regulators PER1/2 and CRY1/2, which attenuate BMAL1/CLOCK activity through a delayed transcription-translation negative feedback loop (TTFL) (Rosensweig and Green, 2020; Laothamatas et al, 2023).

According to the dual repression model of the TTFL (Koike et al, 2012; Ye et al, 2014; Cao et al, 2021), the early repressive phase involves binding of PER/CRY complexes to the BMAL1/CLOCK heterodimer. This interaction, which primarily depends on CRYs binding to the PAS-B domain of CLOCK, leads to displacement of the transcription factor from its target promoters. During this phase of the circadian cycle, the homologs PER1 and PER2, as well as CRY1 and CRY2, perform mechanistically redundant repressor functions, despite differences in their expression phases and mutual binding affinities. In the late repressive phase, following PER protein degradation, CRY1—unlike CRY2—continues to repress BMAL1/CLOCK by associating with CLOCK-PAS-B (Fribourgh et al, 2020) and sequestering the BMAL1 transactivation domain (TAD) (Xu et al, 2015), without interfering with its capacity to bind DNA. As a result, target promoters are dynamically locked in a poised state, marked by the presence of Ser5-phosphorylated RNA polymerase II (Koike et al, 2012). Finally, the model proposes that a new circadian cycle begins when CRY1 is ultimately degraded, allowing BMAL1/CLOCK to regain activity and efficiently reinitiate transcription from these poised promoters. According to this model, repression primarily depends on the stoichiometric sequestration of a sufficient proportion of BMAL1/CLOCK by PER/CRY complexes during the early repressive phase, and by CRY1 alone during the late phase.

**Figure 1:**
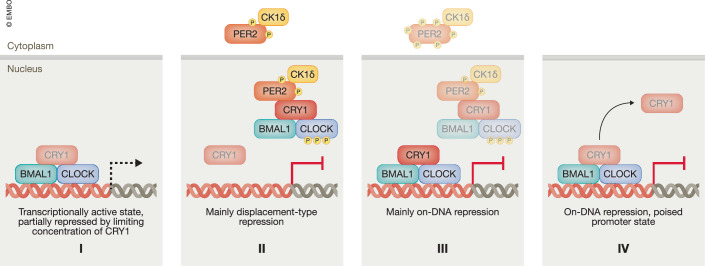
Refined dual-mode repression model of the mammalian circadian clock. CRY1 is present throughout the circadian cycle. I. *Partial repression*: CRY1 partially represses BMAL1/CLOCK even at its circadian peak. It remains active but below the threshold needed for full repression. II. *Off-DNA repression*: PER/CRY complexes displace BMAL1/CLOCK from its promoters, a process dependent on CLOCK phosphorylation by PER-associated CK1δ. III. *Transition to on-DNA repression*: PER becomes hyperphosphorylated, and the nuclear PER:CRY1 ratio decreases due to PER degradation and its cytosolic accumulation in the absence of CRY1. BMAL1/CLOCK is dephosphorylated and rebinds its target promoters. Free CRY1 represses BMAL1/CLOCK without disrupting its DNA-binding ability. IV. *On-DNA repression*: PER2 is degraded. CRY1 continues to repress BMAL1/CLOCK. As CRY1 levels decline, transcription gradually resumes. Although distinct complexes are depicted, specific interactions may occur within a local, dynamic microenvironment around BMAL1/CLOCK binding sites.

In the new study (Smyllie et al, 2025), the authors quantify PER2, CRY1, and BMAL1 within individual cells of organotypic SCN slices. These quantitative measurements were enabled by knock-in mouse lines expressing pairs of fluorescently-tagged proteins—CRY1::mRuby3 with PER2::Venus, or CRY1::mRuby3 with Venus::BMAL1—and a knock-in mouse line harboring a color-switchable PER2::mVenus/mRuby3 fusion, which allowed normalization across expression pairs. Their findings suggest that the dual repression model may warrant refinement regarding its emphasis on stoichiometry-based repression and the clear temporal distinction between repression and activation modes. Since their data originate from a physiologically relevant, robustly oscillating post-mitotic tissue, these findings provide valuable context for interpreting results from cell culture or cell-free systems. While such reductionist approaches are performed under well-defined but often less physiological conditions, the new results allow for direct analysis of molecular interactions and mechanisms underlying discrete steps of the feedback loop (Fig. 1).

Of the three core clock proteins analyzed by the authors of the new study (Smyllie et al, 2025), PER2 was the least stable and exhibited the most pronounced rhythmicity in abundance. By definition, PER2 peaks at circadian time (CT) 12 in the SCN. It declined to ~20% of peak levels by CT 0, confirming strong regulation of PER2 at the levels of synthesis and degradation. Interestingly, around 27% of PER2 protein was cytosolic throughout the circadian cycle. The current clock model does not consider a role of cytosolic PER2 throughout the cycle. CRY1 levels also oscillated markedly but peaked seven hours later than its binding partner PER2, at CT 19. CRY1 reached its trough at CT 7, where its levels were still at approximately 40% of the peak. Unlike PER2, CRY1 was predominantly nuclear, with only ~9% found in the cytosol. At CT12, PER2 and CRY1 were expressed at similar levels but distributed differently between the nucleus and the cytosol. BMAL1 was expressed at the highest levels among the three proteins and was remarkably stable in the SCN. It displayed a rather shallow abundance rhythm, with a peak one hour after CRY1, at about CT 20. At its trough at CT 8, BMAL1 still retained almost 90% of its peak abundance. This minimal fluctuation suggests that BMAL1 is not significantly regulated at the level of protein abundance in this post-mitotic tissue. At the PER2 peak (CT12 in the SCN), BMAL1 was expressed at about 2 to 2.5-fold higher levels than nuclear PER2 or nuclear CRY1, and at CT19, BMAL1 levels were about 1.2-fold higher than the peak levels of CRY1.

The quantification presented here by Smyllie et al raises an important question: are PER/CRY complexes and CRY1 present in the nucleus at sufficient levels to suppress BMAL1/CLOCK activity during the early and late repressive phases—and is CRY1 subsequently cleared efficiently enough to permit full reactivation of BMAL1/CLOCK?

The abundance of CRY1 during the late repressive phase may, in principle, just be sufficient to quantitatively sequester BMAL1/CLOCK. Whether there are enough PER/CRY complexes to displace BMAL1/CLOCK from its target promoters during the early repressive phase remains uncertain, as PER1 and CRY2 levels were not measured. The high-resolution live-cell data of Smyllie et al align well with proteomics data from mouse liver (Narumi et al, 2016), obtained at lower temporal resolution (considering the phase shift between these organs). Based on SCN and extrapolating from liver data, PER/CRY complexes could be present at similar abundance to BMAL1/CLOCK but are likely not much more abundant. Importantly, although excess repressor abundance is a prerequisite for quantitative sequestration of BMAL1/CLOCK, effective repression ultimately depends on whether the concentrations and binding affinities permit saturating complex formation. The nuclear concentrations of the core clock proteins can be estimated from quantitative mass spectrometry (Narumi et al, 2016). Considering the average size of a liver cell nucleus, the concentrations fall in the range of 30–300 nM. PER2 binds the primary pocket of the CRY1 PYR domain with high (8 nM) affinity (Schmalen et al, 2014), suggesting that complexes should form efficiently under physiological conditions. The high fraction of free cytosolic PER2 observed by Smyllie et al, despite excess of CRY1 in the nucleus, may therefore hint at regulation. Preliminary in vitro data suggest that phosphorylation of PER2 by CK1δ disrupts its interaction with CRY1 (Serrano et al, 2025), which could, in addition to PER degradation, contribute to the nuclear clearance of PER during the transition into the late repressive phase.

Binding of BMAL1/CLOCK to PER/CRY or to CRY1 alone is determined by the interaction of the CLOCK PAS-B domain with the secondary binding pocket of CRY (Fribourgh et al, 2020), and to a much lesser extent by weak interaction of CRY with the BMAL1 TAD (Xu et al, 2015). The binding constant for the interaction between the CLOCK PAS-B and CRY1 PYR domains is 65 nM (Fribourgh et al, 2020), in a similar range as the estimated concentrations of these clock proteins. Given the uncertainties in determining both the binding constants and the concentrations of clock proteins, it remains therefore unclear whether PER/CRY and CRY1 can repress BMAL1/CLOCK via saturation binding. However, CLOCK is phosphorylated by CK1δ in a PER/CRY-dependent manner, a modification required for displacement of the BMAL1/CLOCK complex from DNA (Cao et al, 2021). This implies that the repressor complex may act enzymatically, allowing it to function effectively at sub-saturating concentrations. Importantly, this mechanism does not apply to CRY1-mediated repression.

Because the concentrations and binding constants lie within a similar range, binding dynamics may be highly sensitive to even subtle, potentially local changes in concentration. In recent years, it has become clear that transcription factors (TFs) regulate gene expression by transiently clustering with cofactors into dynamic hubs at specific genomic sites, thereby increasing the local concentrations of TFs and their interactors. Circadian repressors may be efficiently recruited to BMAL1/CLOCK hubs. Elevated local concentrations in such microenvironments could promote saturated interactions, while interactions outside these hubs remain minimal. Indeed, recent data from cell culture systems support the notion that substantial fractions of clock proteins may not interact (Xie et al, 2023; Gabriel et al, 2021). Although dynamic complexes—particularly hubs—are challenging to detect in living cells due to their transient nature at physiological temperatures, tissue cooling may stabilize them, enabling their subsequent detection by pull-down assays or native gel electrophoresis (Aryal et al, 2017).

Smyllie et al, report that CRY1 levels remain relatively high even when BMAL1/CLOCK activity peaks, raising the possibility that CRY1 exerts a repressive function even during active circadian transcription. Supporting this, ChIP analyses of mouse liver detected CRY1, though at a low level, alongside initiating RNA polymerase at BMAL1/CLOCK binding sites even during its circadian peak (Koike et al, 2012). Furthermore, recent studies in stable U2OS cell lines show that a PER2-luciferase reporter oscillates at an approximately twofold higher level in CRY1 knockout cells compared to parental cells, and at reduced levels in CRY2 knockout cells (Park et al, 2023) suggesting that CRY1 attenuates BMAL1/CLOCK activity throughout the circadian cycle and that CRY2 antagonizes this repression. Partial repression of BMAL1/CLOCK even at its peak activity may enhance the clock’s responsiveness to entraining or resetting cues.

Last but not least, the authors of this new work (Smyllie et al, 2025) observed that the ratios of TTFL proteins may vary across the SCN. Notably, cells in the phase-leading dorso-medial margin were enriched in CRY1 but showed little PER2 expression. Future studies will hopefully assess whether this pattern is related to the circadian phase waves propagating through the SCN. It will be interesting to determine whether dorso-medial cells express more PER1 instead of PER2, and/or whether they exhibit shorter intrinsic periods when uncoupled using neurotoxins.
